# Oral contraceptives modify the effect of GATA3 polymorphisms on the risk of asthma at the age of 18 years via DNA methylation

**DOI:** 10.1186/1868-7083-6-17

**Published:** 2014-09-19

**Authors:** Kranthi Guthikonda, Hongmei Zhang, Vikki G Nolan, Nelís Soto-Ramírez, Ali H Ziyab, Susan Ewart, Hasan S Arshad, Veeresh Patil, John W Holloway, Gabrielle A Lockett, Wilfried Karmaus

**Affiliations:** 1 Division of Epidemiology, Biostatistics, and Environmental Health, School of Public Health, University of Memphis, Memphis, TN, USA; 2 Department of Epidemiology and Biostatistics, Arnold School of Public Health, University of South Carolina, Columbia, SC, USA; 3 Clinical and Experimental Sciences, Faculty of Medicine, and NIHR Respiratory Biomedical Research Unit, University of Southampton, Southampton, UK; 4 Human Development and Health, Faculty of Medicine, University of Southampton, Southampton, UK; 5 Department of Large Animal Clinical Sciences, Michigan State University, East Lansing, MI, USA; 6 The David Hide Asthma and Allergy Research Centre, Isle of Wight, UK

**Keywords:** *GATA3* gene, DNA methylation, genetic variants, epigenetics, oral contraceptives, age at menarche, asthma, puberty, adolescence, single nucleotide polymorphism, CpG

## Abstract

**Background:**

The prevalence of asthma in girls increases after puberty. Previous studies have detected associations between sex hormones and asthma, as well as between sex hormones and T helper 2 (Th2) asthma-typical immune responses. Therefore, we hypothesized that exogenous or endogenous sex hormone exposure (represented by oral contraceptive pill (OCP) use and early menarche, respectively) are associated with DNA methylation (DNA-M) of the Th2 transcription factor gene, *GATA3,* in turn affecting the risk of asthma in girls, possibly in interaction with genetic variants.

Blood samples were collected from 245 female participants aged 18 years randomly selected for methylation analysis from the Isle of Wight birth cohort, UK. Information on use of OCPs, age at menarche, and concurrent asthma were assessed by questionnaire. Genome-wide DNA-M was determined using the Illumina Infinium HumanMethylation450 beadchip. In a first stage, we tested the interaction between sex hormone exposure and genetic variants on DNA-M of specific cytosine-phosphate-guanine (CpG) sites. In a second stage, we determined whether these CpG sites interact with genetic variants in *GATA3* to explain the risk of asthma.

**Results:**

Interactions between OCP use and seven single nucleotide polymorphisms (SNPs) of *GATA3* were analyzed for 14 CpG sites (stage 1). The interaction between OCP use and SNP rs1269486 was found to be associated with the methylation level of cg17124583 (*P* = 0.002, false discovery rate (FDR) adjusted *P* = 0.04). DNA-M of this same CpG site was also influenced by the interaction between age at menarche and rs1269486 (*P* = 0.0017). In stage 2, we found that cg17124583 modified the association of SNP rs422628 with asthma risk at the age of 18 years (*P* = 0.006, FDR adjusted *P* = 0.04). Subjects with genotype AG showed an increase in average risk ratio (RR) from 0.31 (95% CI: 0.10 to 0.8) to 11.65 (95% CI: 1.71 to 79.5) when methylation level increased from 0.02 to 0.12, relative to genotype AA.

**Conclusion:**

A two-stage model consisting of genetic variants in the *GATA3* gene, OCP use, age at menarche, and DNA-M may explain how sex hormones in women can increase the asthma prevalence after puberty.

## Background

Asthma is a multifactorial disease that is influenced by the interplay between genetic and environmental factors [1]. Studies have shown that the asthma prevalence in girls increases with puberty [1-4]. The mechanism behind this increase is not yet clear, though we propose that endocrine effects may be involved. In addition, asthma and lung function may vary during different phases of the menstrual cycle, further suggesting a role of sex hormones in asthma [5-7]. In addition, it has been reported that both exogenous and endogenous sex hormones influence the occurrence of asthma in young women [8].

Because estrogen and progesterone are known to decrease the contractility of airway smooth muscle, their positive correlation with asthma is more likely driven by their effects on the immune system [9]. Specifically, progesterone stimulates IL-4 production and promotes T helper 2 (Th2) differentiation [10]. The immunological effects of estrogen include increased production of TNF-α by the lungs, increased production of IL-4 by the bone marrow, and thus migration of eosinophils during allergic inflammation [11]. Furthermore, estrogen decreased expression of T-regulatory cells [12], increased expression of IL-5 and IL-13 [13], increased the differentiation of naive CD4^+^ cells into Th2 cells [13], and also increased Th2 responses by augmenting the production of dendritic cells [14]. Hence, estrogen and progesterone may be linked to potential immunological effects and variation in airway responses.

Oral contraceptive pills (OCPs) are exogenous sex hormone preparations used primarily for birth control, but also for irregular menstruation, hirsutism, polycystic ovarian disease, and dysmenorrhea. Some studies find a positive association between OCP use and asthma [15,16], while other studies find the opposite relationship [17,18], and yet other studies identified no significant association [19,20]. In addition, some studies have found early menarche to be associated with the risk of adult asthma [8,15,16], while another study has reported no association [17,18]. Overall, there is a lack of understanding of the association between age at menarche, sex hormones, and asthma.

The transcription factor *GATA3*, located on chromosome 10, encodes a master regulator of Th2 cell differentiation [19] that plays an important role in the production of cytokines [20,21]. A study by Wada *et al,* in a mouse model of asthma, demonstrated increased production of antigen-induced Th2 cytokines in the bronchial lymph node cells of female mice compared to male mice, which was associated with enhanced *GATA3* expression [2], suggesting a possible role for sex in regulating the activity of *GATA3*.

The term epigenetics refers to the changes in phenotype or expression of genes that are not due to changes in the sequence of DNA [22]. Epigenetics is considered to play an important role in regulation and differentiation of T cells and asthma pathogenesis [23,24]. In particular, DNA methylation (DNA-M) may regulate genes associated with asthma and allergy [25]. Some single nucleotide polymorphisms (SNPs) can act as methylation quantitative trait loci (methQTLs) to influence DNA-M at specific CpG sites, and may be conditional on environmental exposure [26-28]. To reflect both the genetic and environmental influences, we call these loci conditional methQTLs.

The role of sex hormones and the potential gender-related activity of *GATA3* in asthma motivated us to study a possible interaction between oral contraceptives and *GATA3* and further its association with asthma. Therefore, we hypothesize that exogenous or endogenous sex hormone exposure in interaction with genetic variants could be associated with DNA-M of *GATA3,* which in turn affects the risk of asthma at the age of 18 years. It is also important to understand whether the change in DNA-M is a cause or a consequence of the disease. To address this issue we use a two-stage model proposed by Karmaus et al., which incorporates both methQTLs and genetic variants [29]. In stage 1, we identify the conditional methQTLs (influenced by the use of OCPs) that may result in a change of the DNA-M of specific CpG sites of the *GATA3* gene. These CpG sites differentially methylated depending on OCP use subsequently may modify the penetrance of certain SNPs, which then are called modifiable genetic variants (modGVs) [30,31]. In stage 2, we evaluate the interaction of differentially methylated CpG sites with modGVs on asthma at the age of 18 years.

Age at menarche is related to changes in endogenous sex hormones and reflects body changes. In girls, the earlier the onset of puberty, the longer the exposure to sex hormones. Hence, we additionally ran our stage 1 model using age at menarche as an alternate indicator of a possible endocrine effect. Agreement between both exposures would further support our hypothesis.

## Results

There were no significant differences in the prevalence of asthma, BMI, smoking at 18 years, maternal history of asthma, socioeconomic status, and median age at menarche between female offspring of the study group of this birth cohort that participated in the 18 year exam (*n* = 660) and those who were randomly selected for the DNA-M analysis (*n* = 245; Table 1). However, OCP missingness was different between the study group at 18 years and the 245 randomly selected girls. The difference is related to more missing information in the female study group at 18 years (*n* = 32, 4.9%) compared to the random selection of 245 with blood samples (*n* = 2, 0.8%). Ignoring missingness, the proportion of OCP use did not differ (*P* = 0.75). The reason for the missingness seems to be parental control at 18 years. In general, participants were interviewed separately in the study center; however, they could also mail the questionnaire or answer some questions on the phone. Fifteen of 16 girls with a mailed questionnaire did not answer the question on OCP use.

**Table 1:** Characteristics of subjects with available methylation data compared to the female participants of the total cohort

	**Total female participants, *n *** **(%)**	**Female participants with DNA-M data, *n *** **(%)**	***P *** **value**
Factors	*n* = 660	*n* = 245	
*Maternal smoking during pregnancy*			
Yes	159 (24.1)	47 (19.2)	
No	498 (75.5)	197 (80.4)	0.29
Missing	7	1 (0.41)	
*Maternal history of asthma*			
Yes	67 (10.2)	30 (12.2)	
No	588 (89.1)	213 (86.9)	0.66
Missing	5 (0.8)	2 (0.82)	
*Asthma at 18 years*			
Yes	128 (19.4)	35 (14.3)	
No	531 (80.5)	210 (85.7)	0.17
Missing	1 (0.2)	0	
*Oral contraceptive use at 18 years*			
Yes	293 (44.4)	117 (47.8)	
No	335 (50.7)	126 (51.4)	0.02
Missing	32 (4.9)	2 (0.8)	
*Smoking at 18 years*			
Yes	192 (29.1)	63 (25.7)	
No	455 (68.9)	181(73.9)	0.13
Missing	13 (2.0)	1 (0.4)	
*Socioeconomic status*			
High	49 (7.4)	22 (9.0)	
Medium	479 (72.6)	182 (74.3)	0.69
Low	94 (14.2)	37 (15.1)	
Missing	38 (5.8)	4 (5.8)	
	*n* (Median; 5%, 95%)	*n* (Median; 5%, 95%)	
*Body mass index at 10 years (kg/m*^*2*^*)*	527 (17.9;14.8, 25.2)	223 (17.9;15.04, 25.3)	0.92
Missing	223	22	
*Body mass index at 18 years (kg/m*^*2*^*)*	499 (22.2;18.2, 32)	240 (22.9;19.05, 32.93)	0.56
Missing	251	5	
*Age at menarche*	631 (13.0;11.0,15.0)	233 (13.0;10.0, 15.0)	0.32
Missing	119	12	

Among the female participants with methylation data, 12.2% had maternal history of asthma, 19.2% had mothers that smoked during pregnancy, 14.3% had asthma at 18 years, 47.8% used OCPs at 18 years (44.4% of the girls in the study group at the 18-year exam). The median age at menarche was found to be 13 years. Use of oral contraceptives and age at menarche in our sample are associated (Wilcoxon test: *P* = 0.001); 62% of the participants with age at menarche ≤11 years, 45.8% of those between 12 and 14 years, and 36% of those with ≥14 years used OCPs.

**Figure 1:**
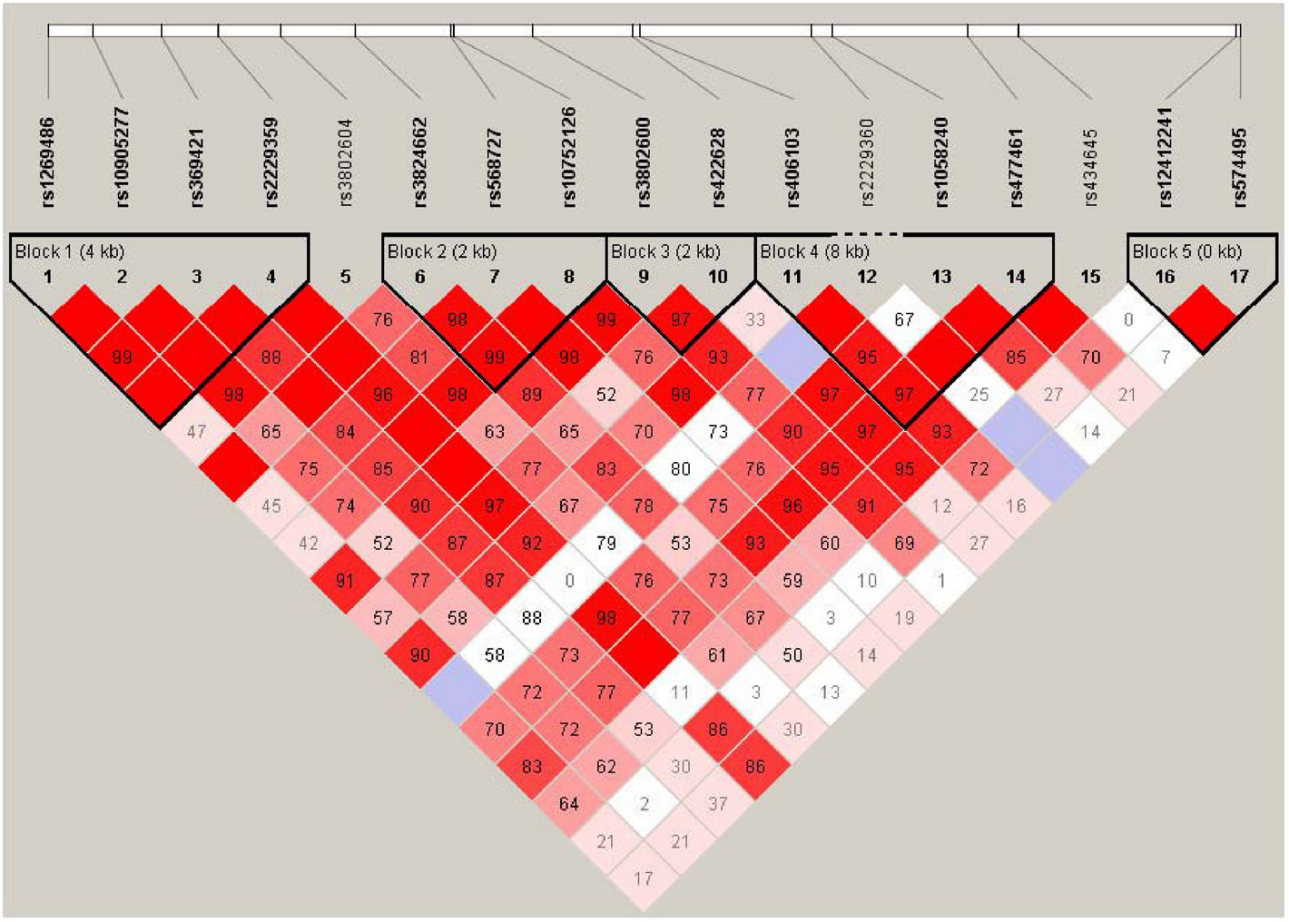
**Linkage disequilibrium of *GATA3 *** **single nucleotide polymorphisms, standard (D’/LOD) color scheme; D’ LD values displayed.**

Of the thirteen *GATA3* SNPs that were genotyped, seven SNPs (rs1269486, rs3802604, rs3824662, rs422628, rs434645, rs12412241, and rs406103) were selected for further analysis since these were uncorrelated (Figure 1). Of the seven SNPs that were analyzed, rs1269486 was located in the promoter, followed by four SNPs (rs3802604, rs3824662, rs422628, and rs406103) in introns, and two SNPs (rs434645, and rs12412241) downstream of the *GATA3* gene (Table 2). The mean methylation levels (β value) of six of the 14 CpG sites of the *GATA3* gene were low (<0.10; Table 3), four were highly methylated (>0.90), while four CpG sites showed wider variation in methylation between individuals with mean methylation between >0.10 and <0.55.

**Table 2:** **Single nucleotide polymorphisms (SNPs) for *GATA3 *** **and their genotypes**

**SNP**	**Position**^**a**^	**Location**	**Genotype**	**Frequency *n *** **(%)**
rs1269486	8096199	Promoter	AA	13 (5.7)
			AG	87 (37.8)
			GG	130 (56.5)
rs3802604	8102272	Intron	AA	88 (38.4)
			AG	103 (45.0)
			GG	130 (56.5)
rs3824662	8104208	Intron	AA	8 (3.4)
			AC	69 (29.6)
			CC	156 (67.0)
rs422628	8111409	Intron	GG	12 (5.1)
			AG	93 (39.9)
			AA	128 (55.0)
rs406103	8111621	Intron (boundary)	AA	13 (5.6)
			AG	78 (33.5)
			GG	142 (60.9)
rs434645	8121451	3’UTR	AA	4 (1.7)
			AG	67 (29.3)
			GG	158 (69.0)
rs12412241	8127139	Downstream	AA	19 (8.1)
			AG	92 (39.7)
			GG	121 (52.1)

^a^Nucleotide position on chromosome 10 based on build GRCh37.p13.

**Table 3:** **Distribution of methylation on CpG sites of *GATA3 *** **gene**

**CpG site**	**Location**	**Position**	**Mean methylation**	**5% value**	**95% value**
cg18599069	5'UTR	8096991	0.06	0.04	0.07
cg10008757	5'UTR	8097183	0.07	0.05	0.09
cg14327531	5'UTR	8097331	0.06	0.04	0.08
cg17124583	Body	8097641	0.05	0.02	0.10
cg19883813^a^	Body	8098005	0.04	0.02	0.08
cg11430077	Body	8099018	0.11	0.05	0.20
cg01255894	Body	8099218	0.06	0.03	0.09
cg10089865	Body	8100286	0.93	0.91	0.95
cg22770911	Body	8101307	0.52	0.44	0.60
cg04492228	Body	8101513	0.19	0.13	0.26
cg17489908	Body	8101566	0.25	0.17	0.34
cg03669298	Body	8102210	0.06	0.04	0.09
cg00463367	Body	8103673	0.20	0.11	0.31
cg04213746	Body	8106003	0.95	0.93	0.96
cg27409129	Body	8111731	0.93	0.92	0.94
cg07989490^b^	3'UTR	8117026	0.95	0.94	0.97

^a^CpG site is not considered for further analysis because of a low methylation level (<5%).

^b^CpG site is not considered for further analysis because of a probe SNP.

In stage 1, after controlling for cell type composition in peripheral blood, the interaction term ‘OCP use × rs1269486’ was found to be associated with differential methylation of cg17124583 (*P* value = 0.002; FDR *P* value = 0.04; Table 4), indicating that this SNP represents a conditional methQTL. OCP users with minor allele (AA) (the difference in a logit scale is -0.86; *P* value = 0.03) and heterozygous (AG) (the difference in a logit scale is -0.57; *P* value = 0.002) genotypes for rs1269486 had lower average methylation than those with the major (GG) genotype. The association was adjusted for potential confounders including socioeconomic status, smoking at 18 years, and BMI at 18 years. However, none of these potential confounders changed the interaction effect by more than 10%.

**Table 4:** **Assessment of interaction of single nucleotide polymorphisms with oral contraceptive use, and with age at menarche on the methylation of the CpG site cg17124583 using linear regression**^**a**^

**Parameter**		**Estimate (Standard error)**		***P *** **value**	
		**Not adjusted for cell type**	**Adjusted for cell type**	**Not adjusted for cell type**	**Adjusted for cell type**^**a**^
OCP use		0.12 (0.11)	0.16 (0.11)	0.28	0.16
rs1269486	AA	0.64 (0.24)	0.68 (0.25)	0.009	0.006
	AG	0.41 (0.12)	042 (0.13)	0.001	0.001
	GG	Reference			
OCP use × rs1269486	AA	-0.72 (0.39)	-0.86 (0.40)	0.06	0.03
	AG	-0.55 (0.18)	-0.57 (0.18)	0.002	0.002
	GG	Reference			
Age at menarche		-0.09 (0.04)	-0.09 (0.04)	0.05	0.06
rs1269486	AA	-5.28 (1.77)	-4.87 (1.78)	0.003	0.006
	AG	-1.12 (0.86)	-1.06 (0.86)	0.19	0.21
	GG	Reference			
Age at menarche × rs1269486	AA	0.45 (0.14)	0.42 (0.14)	0.001	0.003
	AG	0.09 (0.06)	0.09 (0.06)	0.14	0.16
	GG	Reference			

^a^Adjusted for socioeconomic status, smoking at 18 years, BMI at 18 years, and cell mixture of the peripheral blood using the Houseman formula [41].

To replicate the OCP usage model with an alternate indicator for endocrine effects, age at menarche was investigated (Table 4). Indeed, methylation of cg17124583 was differentially methylated by the interaction of same SNP rs1269486 and age at menarche (*P* = 0.0017). In girls with the minor and heterozygous genotypes for rs1269486 methylation levels at cg17124583 were found to be higher if age at menarche was higher. The interaction was statistically significant only in those with the minor genotype (the difference in logit scale is 0.42; *P* value = 0.003). Hence, both OCP use and age at menarche in interaction with rs1269486 were associated with differential methylation of cg17124583.

Interestingly, in a small sample of 34 paired DNA-M measurements, the differentially methylated CpG site cg17124583 show some variability from 10 to 18 years (test for stability: ICC = 0.39, *P* = 0.01) with mean methylation levels of 0.06 and 0.05, respectively. This CpG site shows both stability and variability, but its variance was not explained by OCP use or by age at menarche (data not shown).

**Figure 2:**
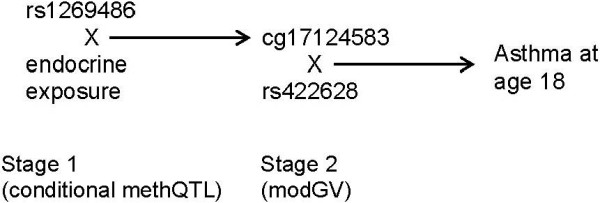
Consecutive assessments of stage 1 (conditional methylation quantitative trail locus) and stage 2 (modifiable genetic variant) assessments.

**Figure 3:**
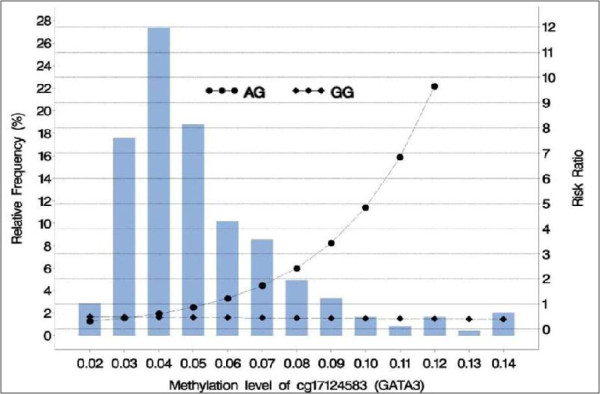
Risk ratio of asthma at 18 years versus methylation at different genotypes of GATA3 rs422628: AG and GG compared to AA [reference].

In the second stage, we analyzed whether methylation of cg17124583 modifies the association between SNPs and asthma at 18 years. We tested the interaction between seven SNPs and the methylation levels of cg17124583 (differentially methylated in stage 1), and its association with asthma at 18 years. We found statistically significant interactions between the SNPs rs434645 and rs422628 with cg17124583 that modify the risk of asthma at 18 years (Table 5). For rs434645, the minor (AA) and heterozygous (AG) genotypes were combined since the direction of effect on methylation was the same for both. Then the statistical association of the interaction of cg1712583 and rs434645 (AA/AG vs. GG) with asthma at 18 years was checked using the common genotype (GG) as the reference. The interaction was found to be significant (*P* = 0.01; Table 5), however, it did not survive multiple testing with FDR. For the SNP rs422628, an additive genetic model was used to compare participants who had the minor (GG) and heterozygous (AG) genotypes, with those who have common (AA) genotype. The interaction term ‘cg17124583 × rs422628’ was found to be statistically significantly associated with asthma in those with the heterozygous genotype after adjusting for multiple comparisons (*P* = 0.006; FDR adjusted *P* = 0.05; Table 5). The consecutive flow of assessments and its results is outlined in Figure 2. The range of DNA-M for cg17124583 was 0.01 to 0.46. Since the number of participants at methylation levels of <0.02 and >0.14 were low, we grouped lower methylation levels into ≤0.02 (*n* = 4) and larger into ≥0.14 (*n* = 9). Descriptively, 157 participants had average methylation levels of 0.05 and less at this CpG, 71 participants had 0.06 to 0.09, and 17 participants had 0.10 to 0.46. For subjects with AG and GG genotypes, we examined the RRs for asthma at different levels of DNA methylation. Here we present RRs for the AG genotype for the following levels: 0.02, 0.04, 0.06, 0.08, 0.10, and 0.12 relative to subjects with AA genotype. For the AG genotype, the corresponding RRs of asthma are 0.31, 0.63, 1.31, 2.71, 5.62, and 11.65 (Figure 3). The respective 95% CI are found in the legend of Figure 3. Figure 3 shows that the relative risk (RR) for the rs422628 AG genotype relative to AA was higher when cg17124583 was more methylated.

**Table 5:** **Log-linear models of interaction between genetic variants (rs434645 and rs422628) with DNA methylation of cg17124583 in the *GATA3 *** **gene on the prevalence of asthma at 18 years**^**a**^

**Single nucleotide and CpG site**	**Genotype**	**Estimate**^**b **^**(log RR)**	**95% CI of log RR**	***P *** **value**
Model for rs434645:				
cg17124583		-20.31		
rs434645	AA and AG	-1.19	-2.61, 0.23	0.10
	GG	Reference		
cg17124583 × rs434645	AA and AG	32.77	7.36, 58.17	0.01
	GG	Reference		
Model for rs422628:				
rs422628	GG	-0.35	-3.58, 2.89	0.83
	AG	-1.91	-3.41, -0.41	0.01
	AA	Reference		
cg17124583 × rs422628	GG	-8.62	-71.11, 53.86	0.79
	AG	36.41	10.67, 62.14	0.006
	AA	Reference		

^a^Adjusted for socioeconomic status, maternal smoking during pregnancy, smoking at age 18, and BMI at age 18.

^b^The estimate needs to be exponentiated to calculate the risk ratio. In addition, to estimate the risk ratio due to the interaction, we need to take the two main effects and the interaction effect into account. The information is provided in Figure 3.

## Discussion

Of the 14 CpGs and the seven SNPs that were analyzed, we identified a conditional methQTL (rs1269486) interacting with OCP usage and with age at menarche leading to a differential DNA-M of cg17124583 (Figure 2). The same differentially methylated CpG site cg17124583 in interaction with another SNP rs422628 (modGV) was found to modify the association of asthma at 18 years. This association remained statistically significant after adjusting for multiple comparisons using FDR. To our knowledge, this is the first study to identify those SNPs in the *GATA3* gene that in interaction with OCP use, and with age at menarche, are associated with differential methylation of *GATA3* CpG sites and consecutively with asthma.

Although the CpG site cg17124583 is located 13,768 base pairs away from rs422628, we can see that the risk of asthma is modulated by this CpG site. It is possible that rs422628 is in linkage disequilibrium with another genetic variant, which is responsible for the functional effect on asthma risk and is adjacent to cg17124583, as has been previously observed for another gene [32].

The probability of a selection bias seems to be negligible as the study participants were randomly selected for the DNA-M analysis and for all but one variable there were no significant differences between the study population and the cohort girls who participated at 18 years. However, the proportion of missing information about the use of oral contraceptives at 18 years of age was higher in female cohort members. This was likely due to parental control, since nearly all girls whose questionnaire was mailed had missing information. We do not consider that the bias of parental control biases our results.

As the information on the use of OCPs is self-reported by the participants, there is a possibility of a misclassification. However, previous studies have shown high agreement between questionnaire data and medical records for any OCP use, current use, and time since first use [33,34]. In addition, since age at menarche is an important event in a women’s life, thus, misclassifications are unlikely [35]. As the women were neither aware of their SNPs nor the methylation status, any recall bias would result in a non-differential misclassification and likely underestimate the true association. We repeated the analyses with a different exposure marker for endocrine effects, namely age at menarche, which showed a similar result. It therefore seems highly likely that the significant effects we observe on asthma risk represent an authentic link to endocrine events via differential methylation of the *GATA3* gene.

The DNA-M in our study was obtained using the Illumina Infinium HumanMethylation450 beadchip array, which is demonstrated to have high validity and high reproducibility [36]. As DNA-M can be tissue-specific, it is also important to consider whether the DNA-M obtained from peripheral blood represented methylation profiles in other tissues. This issue is currently under debate [37-40]. In addition, peripheral blood leukocytes represent a mixture of cells [41]. Using CpG site information, we estimated the relative contribution of cell type composition in peripheral blood using the Houseman approach [41]. The estimated cell type composition had only a minor influence on the DNA-M of *GATA3* CpG sites (Table 4), suggesting that differences in the proportions of different leukocyte do not underlie the effects reported here.

In the regression models, we observed that, although the main effects of OCP use, age at menarche, and SNPs were not significantly associated with DNA-M of cg17124583, their interactions were found to be significantly associated even after penalizing for multiple testing. Similarly, no main effects were seen for the association of OCP use, cg17124583, and the SNPs on the risk for asthma at 18 years. However, the interaction of the SNP and DNA-M were found to be statistically significant. The importance of genome-epigenome interactions in disease is increasingly recognized [42]. For example, DNA-M at the *IL4R* locus interacts with a local SNP to increase the RR of asthma much more dramatically than does either genotype or methylation alone [43]. Likewise DNA-M and genotype at the *IL13* locus interact to influence lung function [44]. It is therefore of great importance to consider not only the disease risk imparted by the genome sequence, but how this is modified by DNA-M, which itself by be affected by environmental exposures.

Asthma being considered mainly a ‘Th2 disease’, we focused on the *GATA3* gene because it is known to be the master regulator of Th2 cell differentiation [19] and has been linked to endocrine responses [45]. Estrogen is an immune modulator and is known to stimulate the production of Th2 cytokines, which include IL-4, IL-5, and IL-13 [11,13]. Our findings show that OCP use modifies the DNA-M of *GATA3* gene. We speculate that OCPs, which contain estrogen and progesterone [46,47], may influence Th2 cytokine production via the differential methylation of *GATA3* gene. Similar findings are seen with age at menarche altering the DNA-M of *GATA3* gene. Statistically, although early age at menarche is related to use of OCPs, the two variables are not in complete agreement and seem to measure different features. Age at menarche is related to endogenous sex hormones [48], whereas OCPs are exogenous sex hormones. We believe that the agreement of our stage 1 findings between OCP use and age at menarche provides credence to our results. Our two-stage model suggests a potential pathway in which sex hormone-related exposures such as OCP use and age at menarche alter the DNA-M within *GATA3* to subsequently affect the risk for asthma in girls at 18 years. We believe that using the two-stage model prevents reverse associations, namely that asthma initiates changes of CpG sites. In the first stage, cg17124583 was the only CpG site selected due to its relation with the interaction term of OCP use with one genetic variant (rs1269486) of the GATA3 gene and corroborated with age at menarche. Then only this CpG site was tested for an association with asthma at 18 years. However, it is not likely, but still possible that three variables (oral contraceptive use, asthma, and rs1269486) interacted in concert to influence the methylation of cg17124583.

A limitation of our study is that the RRs at methylation levels larger than 9% are only based on a limited number of individuals (*n* = 17). Another limitation is the lack of availability of the information on the type of OCP (estrogen/progesterone only pills or a combined pill), and length of time on the OCP which can further help to elucidate the role of either estrogen/progesterone or both in DNA methylation, genetic polymorphisms, and asthma.

## Conclusions

This study represents the first report of an interaction of genetic variation and DNA-M of *GATA3* on the risk for asthma at 18 years, which is modified by the use of OCP and age at menarche. The findings suggest a potential pathway in which OCP exposure and age at menarche, presumably via sex hormones, can alter the DNA-M of a *GATA3* CpG site, which subsequently, in conjunction with genetic variants, influences the risk of asthma at 18 years. These findings provide a possible explanation for the increase in asthma prevalence in girls/women after puberty. Our results should motivate other researchers to search for interactions between genetic variants, sex hormones, and DNA-M.

## Methods

### Study design and population

A whole population birth cohort was established in the Isle of Wight, UK in 1989 to prospectively study the natural history and etiology of asthma and allergic conditions. The local research ethics committee (NRES Committee South Central - Hampshire B) approved the study and written informed consent was obtained from 1,456 children (January 1989 to February 1990), who were followed up at 1, 2, 4, 10, and 18 years. This Caucasian birth cohort has been described in detail elsewhere [49]. Questionnaires were completed for each child at every follow-up. Blood or saliva samples were collected at the ages of 10 and 18 years for genetic analysis.

### Exposures

Information on OCP use was collected at 18 years. The question was: ‘Are you on the contraceptive pill?’ Age at menarche was assessed using the National Institute of Child and Human Development (NICHD) questionnaire from the Study of Early Child Care and Youth Development, which is based on the Pubertal Development Scale (PDS) method [50]. Among other questions on pubertal signs, the questionnaire asked: ‘How old were you when you started to menstruate?’

### Outcome

Asthma information was collected using the International Study of Asthma and Allergies in Childhood (ISAAC) questionnaire [51]. The questions for assessing asthma were as follows: ‘History of physician diagnosed asthma?’, ‘Wheezing or whistling in the chest in the last 12 months?’ and ‘Asthma treatment in the last 12 months?’ Based on the answers to these questions, asthma at 18 years was defined by physician diagnosis of asthma plus current symptoms and/or currently on asthma medication.

### Genotyping

Genomic DNA was isolated from blood samples by using QIAamp DNA Blood Kits (Qiagen, Valencia, CA, USA) or the ABI PRISM 6100 Nucleic Acid PrepStation (Applied Biosystems, Foster City, CA, USA). In some cases genomic DNA was isolated from saliva using Oragene DNA Self Collection Kits (DNA Genotek, Ottawa, ON, Canada). Polymorphisms in the *GATA3* gene were examined using the SNPper and Applied Biosystems databases. Genotyping was conducted by fluorogenic 5’ nuclease chemistry PCR using Assays on Demands kits cycled on a 7900HT Sequence Detection System (Applied Biosystems, Foster City, CA, USA), or biotin-streptavidin-based pyrosequencing performed on PSQ-6 instrumentation (Biotage AB, Uppsala, Sweden). SNPs (*n* = 17) that tagged the *GATA3* gene were identified using a tagger implemented in Haploview 4.2 using Caucasian Hapmap data, including 10 kb upstream and downstream of the *GATA3* gene [52]. Estimates of linkage disequilibrium (LD) between SNPs were calculated using D’ and r^2^. An r^2^ value of 0.85 was the threshold for tagging, and seven SNPs were selected (1 SNP from each of the 5 haplotype blocks and 2 SNPs that did not have strong linkage disequilibrium with other SNPs, Figure 1).

### DNA Methylation

Stored blood samples collected at 10 and 18 years were available on the Isle of Wight, UK. For the measurement of DNA methylation at 18 years in girls, the team in the United States provided a list of 245 random identification numbers to be selected from the samples on the Isle of Wight, UK. Then for additional DNA methylation analyses of samples when these 245 women were 10 years of age, we randomly selected 34 blood samples with 16 girls with asthma and 18 girls without. DNA methylation was assessed using Illumina Infinium HumanMethylation450 BeadChips (Illumina, Inc, SanDiego, CA, USA). The 18-year samples were processed in one batch. In addition, DNA methylation data were available for a sample of 34 girls at 10 years of age processed in another batch. DNA from blood samples was extracted for methylation arraying using a salting out procedure. One microgram of DNA was bisulfite-treated for cytosine to thymine conversion using the EZ 96-DNA methylation kit (Zymo Research, CA, USA), following the manufacturer’s standard protocol. Arrays were processed using a standard protocol as described elsewhere [53]. The Bead Chips were scanned using a Bead Station, and the methylation level (beta (β) value) was calculated for each queried CpG locus using Methylation module of GenomeStudio software.

### Covariates

Maternal history of asthma and maternal smoking during pregnancy was assessed by a questionnaire administered after birth. Information about the child’s active smoking status and body mass index (BMI) was collected from the 18-year questionnaire and anthropometric measurements conducted at the age of 18 years. Also assessed was ‘family social status cluster’, which is a composite variable derived from a combination of family income, parental occupation (socioeconomic status), and number of children in a child’s bedroom [54].

In addition, since DNA methylation found in peripheral blood cells depends on cell types, we adjusted all stage 1 models for cell mixture using the method proposed by Houseman et al [41]. This method identifies CpGs within differentially methylated regions known to distinguish six types of white blood cells and then utilizes β values at these CpGs to predict the proportions of CD8+ T-cells, CD4+ T-cells, natural killer cells, B-cells, monocytes, and granulocytes for each blood sample. The rationale to use this method for stage 1 is to estimate the change in DNA-M that is due to differential methylation but not due to a change in peripheral blood cell. Once we have identified such differential DNA-M, in stage 2 we are more interested in the concert of cells and their methylation level on the outcome asthma.

### Statistical analysis

Preprocessing of the DNA-M data was undertaken using the IMA [55] package implemented in the R statistical computing package [56]. To identify tag-SNPs, LD between SNPs was calculated using D’ and r^2^[57] and they were tested for Hardy-Weinberg equilibrium using Haploview 3.2 software [52]. DNA-M levels were quantified using β values that present the proportion of methylated (M) over the sum of methylated and unmethylated (U) allele intensities (β = M/[c + M + U]), with c being a constant to prevent dividing by zero [58]. As the β value method has severe heteroscedasticity, it is recommended to use M-values (logit-transformed β values) for differential methylation analysis [59]. A logit transformation was employed for all β values to normalize their distribution. To assess whether the subset population (*n* = 245) represents the total cohort of girls at 18 years, χ^2^ tests were used.

In this study, 16 CpG sites that spanned the *GATA3* gene were analyzed, out of which one CpG site was removed due to the presence of a probe SNP. A probe SNP is a single nucleotide polymorphism in the probe of 50 base-pairs used to determine the location of methylated CpG site. A SNP in the 50 base-pair probe may interfere with the DNA-M measurement. A second CpG site had an average methylation level <0.05, we removed this site from analysis as CpGs that are either very highly (>0.95) or very lowly (<0.05) methylated have too little variance that can be explained statistically. We added analyses on whether chip and positions had an influence on the M-values in the 245 samples. Neither chip nor positions showed significant effects or any substantial changes. In addition, in 34 female participants stability of DNA-M in blood between 10 and 18 years was estimated using intraclass correlation coefficients (ICCs).

The aim of the first stage of the two-stage model was to detect CpG sites that were affected by an interaction of SNPs and OCP usage. We ran linear regression models, in which each of the 14 CpG sites were modeled against seven SNPs, each interacting with OCP use. Since we performed 98 tests (14 × 7) we adjusted for multiple testing by controlling the overall false discovery rate (FDR; overall FDR = 0.05) [60].

Focusing on the CpG sites with significant interactions with OCPs, we then reran the analyses of stage 1 using age at menarche as exposure to determine if similar associations occurred with this marker of endocrine changes. If statistically significant associations are observed for both sex hormone exposures, then this strengthens the evidence that the association is related to sex hormones.

In the second stage model with asthma as the dependent variable, we used log-linear models (GENMOD procedure in SAS 9.3) to estimate statistical interactions between the methylation levels of CpG sites selected in stage 1 and *GATA3* SNPs on the risk for asthma at age 18 years. These models included the following potential confounders: maternal history of asthma, maternal smoking during pregnancy, BMI at 18 years, smoking at 18 years, and socioeconomic status. Those confounders that changed the association of interest by 10% or more were retained as confounders in the final model. All hypotheses tested were corrected for multiple testing using the FDR. The statistical analyses were performed using the SAS statistical package (version 9.3; SAS Institute, Cary, NC, USA).

## Abbreviations

BMI: Body mass index; CI: Confidence Interval; CpG: Cytosine-phosphate-guanine dinucleotide; DNA-M: DNA methylation; FDR: False discovery rate; ICC: Intraclass correlation coefficient; ISAAC: International Study of Asthma and Allergies in Childhood; LD: Linkage disequilibrium; MethQTL: Methylation quantitative trait locus; ModGV: Modifiable genetic variant; OCP: Oral Contraceptive Pill; RR: Risk Ratio; SNP: Single nucleotide polymorphisms; Th: T helper; 5'UTR: Five prime untranslated region; 3'UTR: Three prime untranslated region.

## Competing interests

The authors declare that they have no competing interests.

## Authors’ contributions

KG conducted the statistical analysis, interpreted the data, and drafted the manuscript. JH supervised the assessment of the DNA methylation and revised the manuscript. HZ directed the statistical analysis and aided in their interpretation and the final editing. SE selected and measured the single nucleotide polymorphisms and contributed to funding acquisition and the manuscript. NSR, GAL, and AHZ helped in conducting statistical analysis and editing of the manuscript. VGN aided in the interpretation of data, critical revision and final editing of the manuscript. HA and VKP were responsible for cohort assessments and asthma phenotype data and sample collection and provided critical revision and final editing of the manuscript. WK designed the study, reviewed the data quality, helped with statistical analyses, and revised the manuscript. All authors read and approved the final manuscript.

## References

[B1] Tollefsen E Langhammer A Romundstad P Bjermer L Johnsen R Holmen TL Female gender is associated with higher incidence and more stable respiratory symptoms during adolescence Respir Med 2007 101 896 902 17084607 10.1016/j.rmed.2006.09.022

[B2] Wada K Okuyama K Ohkawara Y Takayanagi M Ohno I Gender differences in transcriptional regulation of IL-5 expression by bronchial lymph node cells in a mouse model of asthma Respirology 2010 15 629 635 20337994 10.1111/j.1440-1843.2010.01721.x

[B3] Soto-Ramirez N Ziyab AH Karmaus W Zhang H Kurukulaaratchy RJ Ewart S Arshad SH Epidemiologic methods of assessing asthma and wheezing episodes in longitudinal studies: measures of change and stability J Epidemiol 2013 23 399 410 23994864 10.2188/jea.JE20120201 PMC3834276

[B4] Postma DS Gender differences in asthma development and progression Gender Medicine 2007 4 S133 S146 18156099 10.1016/s1550-8579(07)80054-4

[B5] Tam A Morrish D Wadsworth S Dorscheid D Man SF Sin DD The role of female hormones on lung function in chronic lung diseases BMC Womens Health 2011 11 24 21639909 10.1186/1472-6874-11-24 PMC3129308

[B6] Siroux V Curt F Oryszczyn MP Maccario J Kauffmann F Role of gender and hormone-related events on IgE, atopy, and eosinophils in the Epidemiological Study on the Genetics and Environment of Asthma, bronchial hyperresponsiveness and atopy J Allergy Clin Immunol 2004 114 491 498 15356546 10.1016/j.jaci.2004.05.027

[B7] Balzano G Fuschillo S Melillo G Bonini S Asthma and sex hormones Allergy 2001 56 13 20 11167347 10.1034/j.1398-9995.2001.00128.x

[B8] Salam MT Wenten M Gilliland FD Endogenous and exogenous sex steroid hormones and asthma and wheeze in young women J Allergy Clin Immunol 2006 117 1001 1007 16675325 10.1016/j.jaci.2006.02.004

[B9] Haggerty CL Ness RB Kelsey S Waterer GW The impact of estrogen and progesterone on asthma Ann Allergy Asthma Immunol 2003 90 284 291 quiz 291-283, 347 12669890 10.1016/S1081-1206(10)61794-2

[B10] Piccinni MP Giudizi MG Biagiotti R Beloni L Giannarini L Sampognaro S Parronchi P Manetti R Annunziato F Livi C Progesterone favors the development of human T helper cells producing Th2-type cytokines and promotes both IL-4 production and membrane CD30 expression in established Th1 cell clones J Immunol 1995 155 128 133 7541410

[B11] de Oliveira AP Domingos HV Cavriani G Damazo AS Dos Santos Franco AL Oliani SM Oliveira-Filho RM Vargaftig BB Cellular recruitment and cytokine generation in a rat model of allergic lung inflammation are differentially modulated by progesterone and estradiol Am J Physiol Cell Physiol 2007 293 C1120 C1128 17634417 10.1152/ajpcell.00286.2006

[B12] Arruvito L Sanz M Banham AH Fainboim L Expansion of CD4(+)CD25(+) and FOXP3(+) regulatory T cells during the follicular phase of the menstrual cycle: Implications for human reproduction Journal of Immunology 2007 178 2572 2578 10.4049/jimmunol.178.4.2572 17277167

[B13] Cai Y Zhou J Webb DC Estrogen stimulates Th2 cytokine production and regulates the compartmentalisation of eosinophils during allergen challenge in a mouse model of asthma Int Arch Allergy Immunol 2012 158 252 260 22398379 10.1159/000331437

[B14] Uemura Y Liu TY Narita Y Suzuki M Matsushita S 17 beta-Estradiol (E2) plus tumor necrosis factor-alpha induces a distorted maturation of human monocyte-derived dendritic cells and promotes their capacity to initiate T-helper 2 responses Human Immunology 2008 69 149 157 18396206 10.1016/j.humimm.2008.01.017

[B15] Macsali F Real FG Plana E Sunyer J Anto J Dratva J Janson C Jarvis D Omenaas ER Zemp E Wjst M Leynaert B Svanes C Early age at menarche, lung function, and adult asthma Am J Respir Crit Care Med 2011 183 8 14 20732985 10.1164/rccm.200912-1886OC

[B16] Al-Sahab B Hamadeh MJ Ardern CI Tamim H Early Menarche Predicts Incidence of Asthma in Early Adulthood American Journal of Epidemiology 2011 173 S293 S293 10.1093/aje/kwq324 21036953

[B17] Jartti T Saarikoski L Jartti L Lisinen I Jula A Huupponen R Viikari J Raitakari OT Obesity, adipokines and asthma Allergy 2009 64 770 777 19210351 10.1111/j.1398-9995.2008.01872.x

[B18] Burgess JA Walters EH Byrnes GB Giles GG Jenkins MA Abramson MJ Hopper JL Dharmage SC Childhood adiposity predicts adult-onset current asthma in females: a 25-yr prospective study Eur Respir J 2007 29 668 675 17251231 10.1183/09031936.00080906

[B19] Zheng WP Flavell RA The transcription factor GATA-3 is necessary and sufficient for Th2 cytokine gene expression in CD4 T cells Cell 1997 89 587 596 9160750 10.1016/s0092-8674(00)80240-8

[B20] Zhu J Min B Hu-Li J Watson CJ Grinberg A Wang Q Killeen N Urban JF Jr Guo L Paul WE Conditional deletion of Gata3 shows its essential function in T(H)1-T(H)2 responses Nat Immunol 2004 5 1157 1165 15475959 10.1038/ni1128

[B21] Lee HJ Takemoto N Kurata H Kamogawa Y Miyatake S O'Garra A Arai N GATA-3 induces T helper cell type 2 (Th2) cytokine expression and chromatin remodeling in committed Th1 cells J Exp Med 2000 192 105 115 10880531 10.1084/jem.192.1.105 PMC1887713

[B22] Egger G Liang GN Aparicio A Jones PA Epigenetics in human disease and prospects for epigenetic therapy Nature 2004 429 457 463 15164071 10.1038/nature02625

[B23] Runyon RS Cachola LM Rajeshuni N Hunter T Garcia M Ahn R Lurmann F Krasnow R Jack LM Miller RL Swan GE Kohli A Jacobson AC Nadeau KC Asthma discordance in twins is linked to epigenetic modifications of T cells Plos One 2012 7 e48796 23226205 10.1371/journal.pone.0048796 PMC3511472

[B24] Kumar RK Hitchins MP Foster PS Epigenetic changes in childhood asthma Dis Model Mech 2009 2 549 553 19892885 10.1242/dmm.001719

[B25] Kim EG Shin HJ Lee CG Park HY Kim YK Park HW Cho SH Min KU Cho ML Park SH Lee CW DNA methylation and not allelic variation regulates STAT6 expression in human T cells Clin Exp Med 2010 10 143 152 19949830 10.1007/s10238-009-0083-8

[B26] Shoemaker R Deng J Wang W Zhang K Allele-specific methylation is prevalent and is contributed by CpG-SNPs in the human genome Genome Research 2010 20 883 889 20418490 10.1101/gr.104695.109 PMC2892089

[B27] Bell JT Pai AA Pickrell JK Gaffney DJ Pique-Regi R Degner JF Gilad Y Pritchard JK DNA methylation patterns associate with genetic and gene expression variation in HapMap cell lines Genome Biol 2011 12 R10 21251332 10.1186/gb-2011-12-1-r10 PMC3091299

[B28] Hellman A Chess A Extensive sequence-influenced DNA methylation polymorphism in the human genome Epigenetics Chromatin 2010 3 11 20497546 10.1186/1756-8935-3-11 PMC2893533

[B29] Karmaus W Ziyab AH Everson T Holloway JW Epigenetic mechanisms and models in the origins of asthma Curr Opin Allergy Clin Immunol 2013 13 63 69 23242116 10.1097/ACI.0b013e32835ad0e7 PMC3952069

[B30] Malousi A Kouidou S DNA hypermethylation of alternatively spliced and repeat sequences in humans Molecular Genetics and Genomics 2012 287 631 642 22740315 10.1007/s00438-012-0703-y PMC3407362

[B31] Oberdoerffer S A conserved role for intragenic DNA methylation in alternative pre-mRNA splicing Transcription 2012 3 106 109 22771943 10.4161/trns.19816 PMC3616078

[B32] Grundberg E Meduri E Ling JKS Hedman AK Keildson S Buil A Busche S Yuan W Nisbet J Sekowska M Wilk A Barrett A Small KS Ge B Caron M Shin SY Lathrop M Dermitzakis ET McCarthy MI Spector TD Bell JT Deloukas P Multiple Tissue Human Expression Resource Consortium Global analysis of DNA methylation variation in adipose tissue from twins reveals links to disease-associated variants in distal regulatory elements (vol 93, pg 876, 2013) Am J Hum Genet 2013 93 1158 1158 10.1016/j.ajhg.2013.10.004 PMC3824131 24183450

[B33] Bean JA Leeper JD Wallace RB Sherman BM Jagger H Variations in the reporting of menstrual histories Am J Epidemiol 1979 109 181 185 425957 10.1093/oxfordjournals.aje.a112673

[B34] Norell SE Boethius G Persson I Oral contraceptive use: interview data versus pharmacy records International Journal of Epidemiology 1998 27 1033 1037 10024199 10.1093/ije/27.6.1033

[B35] Must A Phillips SM Naumova EN Blum M Harris S Dawson-Hughes B Rand WM Recall of early menstrual history and menarcheal body size: After 30 years, how well do women remember? American Journal of Epidemiology 2002 155 672 679 11914195 10.1093/aje/155.7.672

[B36] Bibikova M Barnes B Tsan C Ho V Klotzle B Le JM Delano D Zhang L Schroth GP Gunderson KL Fan JB Shen R High density DNA methylation array with single CpG site resolution Genomics 2011 98 288 295 21839163 10.1016/j.ygeno.2011.07.007

[B37] Talens RPBD Tobi EW Kremer D Jukema JW Willemsen G Putter HSP Heijmans BT Variation, patterns, and temporal stability of DNA methylation: considerations for epigenetic epidemiology FASEB J 2010 24 3135 3144 20385621 10.1096/fj.09-150490

[B38] Terry MB Delgado-Cruzata L Vin-Raviv N Wu HC Santella RM DNA methylation in white blood cells Association with risk factors in epidemiologic studies Epigenetics 2011 6 828 837 21636973 10.4161/epi.6.7.16500 PMC3154425

[B39] Heijmans BT Mill J Commentary: The seven plagues of epigenetic epidemiology International Journal of Epidemiology 2012 41 74 78 22269254 10.1093/ije/dyr225 PMC3304528

[B40] Ma B Wilker EH Willis-Owen SA Byun HM Wong KC Motta V Baccarelli AA Schwartz J Cookson WO Khabbaz K Mittleman MA Moffatt MF Liang L Predicting DNA methylation level across human tissues Nucleic Acids Res 2014 42 3515 3528 24445802 10.1093/nar/gkt1380 PMC3973306

[B41] Houseman EA Accomando WP Koestler DC Christensen BC Marsit CJ Nelson HH Wiencke JK Kelsey KT DNA methylation arrays as surrogate measures of cell mixture distribution BMC Bioinformatics 2012 13 86 22568884 10.1186/1471-2105-13-86 PMC3532182

[B42] Cooper DN Krawczak M Polychronakos C Tyler-Smith C Kehrer-Sawatzki H Where genotype is not predictive of phenotype: towards an understanding of the molecular basis of reduced penetrance in human inherited disease Hum Genet 2013 132 1077 130 23820649 10.1007/s00439-013-1331-2 PMC3778950

[B43] Soto-Ramirez N Arshad SH Holloway J Zhang H Schauberger E Ewart S Patil V Karmaus W The interaction of genetic variants and DNA methylation of the interleukin-4 receptor gene increase the risk of asthma at age 18 years Clinical Epigenetics 2013 5 1 23286427 10.1186/1868-7083-5-1 PMC3544634

[B44] Patil VK Holloway JW Zhang H Soto-Ramirez N Ewart S Arshad SH Karmaus W Interaction of prenatal maternal smoking, interleukin 13 genetic variants and DNA methylation influencing airflow and airway reactivity Clin Epigenetics 2013 5 22 24314122 10.1186/1868-7083-5-22 PMC3892084

[B45] Parikh P Palazzo JP Rose LJ Daskalakis C Weigel RJ GATA-3 expression as a predictor of hormone response in breast cancer J Am Coll Surg 2005 200 705 710 15848360 10.1016/j.jamcollsurg.2004.12.025

[B46] Salem ML Estrogen, a double-edged sword: modulation of TH1- and TH2-mediated inflammations by differential regulation of TH1/TH2 cytokine production Curr Drug Targets Inflamm Allergy 2004 3 97 104 15032646 10.2174/1568010043483944

[B47] Miyaura H Iwata M Direct and indirect inhibition of Th1 development by progesterone and glucocorticoids Journal of Immunology 2002 168 1087 1094 10.4049/jimmunol.168.3.1087 11801642

[B48] Apter D Reinila M Vihko R Some Endocrine Characteristics of Early Menarche, a Risk Factor for Breast-Cancer, Are Preserved into Adulthood International Journal of Cancer 1989 44 783 787 10.1002/ijc.2910440506 2511157

[B49] Arshad SH Hide DW Effect of environmental factors on the development pf allergic disorders in infancy J Allergy Clin Immun 1992 90 235 241 1500628 10.1016/0091-6749(92)90077-f

[B50] Brooks-Gunn J Warren MP Rosso J Gargiulo J Validity of self-report measures of girls' pubertal status Child Dev 1987 58 829 41 3608653

[B51] Asher MI Keil U Anderson HR Beasley R Crane J Martinez F Mitchell EA Pearce N Sibbald B Stewart AW Strachan D Weiland SK Williams HC International study of asthma and allergies in childhood (ISAAC): rationale and methods Eur Respir J 1995 8 483 491 7789502 10.1183/09031936.95.08030483

[B52] Barrett JC Fry B Maller J Daly MJ Haploview: analysis and visualization of LD and haplotype maps Bioinformatics 2005 21 263 265 15297300 10.1093/bioinformatics/bth457

[B53] Bibikova MFJ GoldenGate assay for DNA methylation profiling Methods Mol Biology 2009 507 149 163 10.1007/978-1-59745-522-0_12 18987813

[B54] Ogbuanu IU Karmaus W Arshad SH Kurukulaaratchy RJ Ewart S Effect of breastfeeding duration on lung function at age 10 years: a prospective birth cohort study Thorax 2009 64 62 66 19001004 10.1136/thx.2008.101543 PMC2630423

[B55] Wang DYL Hu Q Sucheston LE Higgins MJ Ambrosone CB IMA: an R package for high-throughput analysis of Illumina's 450 K Infinium methylation data Bioinformatics 2012 28 729 730 22253290 10.1093/bioinformatics/bts013 PMC3289916

[B56] R: A language and environment for statistical computing. R Foundation for Statistical Computing [http://www.R-project.org/]

[B57] Hill WG Robertson A The effect of linkage on limits to artificial selection (Reprinted) Genet Res 2007 89 311 336 18976519 10.1017/S001667230800949X

[B58] Kuan PFWS Zhou X Chu H A statistical framework for Illumina DNA methylation arrays Bioinformatics 2010 26 2849 2855 20880956 10.1093/bioinformatics/btq553 PMC3025715

[B59] Du P Zhang X Huang CC Jafari N Kibbe WA Hou L Lin SM Comparison of Beta-value and M-value methods for quantifying methylation levels by microarray analysis BMC Bioinformatics 2010 11 587 21118553 10.1186/1471-2105-11-587 PMC3012676

[B60] Benjamini Y Hochberg Y Controlling the False Discovery Rate - a Practical and Powerful Approach to Multiple Testing J Roy Stat Soc B Met 1995 57 289 300

